# Psychological therapies for depression and cardiovascular risk: evidence from national healthcare records in England

**DOI:** 10.1093/eurheartj/ehad188

**Published:** 2023-04-18

**Authors:** Céline El Baou, Roopal Desai, Claudia Cooper, Natalie L Marchant, Steve Pilling, Marcus Richards, Rob Saunders, Joshua E J Buckman, Elisa Aguirre, Amber John, Joshua Stott

**Affiliations:** Adapt Lab, Research Department of Clinical, Educational and Health Psychology, UCL, 1-19 Torrington Place, Camden, London WC1E 7HB, UK; Adapt Lab, Research Department of Clinical, Educational and Health Psychology, UCL, 1-19 Torrington Place, Camden, London WC1E 7HB, UK; Centre for Psychiatry and Mental Health, Wolfson Institute of Population Health, Queen Mary University of London, London, UK; Tower Hamlets Memory Service, East London NHS Foundation Trust, London, UK; Marchant Lab, Division of Psychiatry, UCL, London, UK; Centre for Outcomes Research and Effectiveness, Research Department of Clinical, Educational and Health Psychology, UCL, London, UK; Camden & Islington NHS Foundation Trust, St Pancras Hospital, London, UK; MRC Unit for Lifelong Health and Ageing at UCL, UCL, London, UK; Adapt Lab, Research Department of Clinical, Educational and Health Psychology, UCL, 1-19 Torrington Place, Camden, London WC1E 7HB, UK; Centre for Outcomes Research and Effectiveness, Research Department of Clinical, Educational and Health Psychology, UCL, London, UK; Centre for Outcomes Research and Effectiveness, Research Department of Clinical, Educational and Health Psychology, UCL, London, UK; iCope—Camden & Islington NHS Foundation Trust, St Pancras Hospital, London, UK; North East London NHS Foundation Trust (NELFT), London, UK; Adapt Lab, Research Department of Clinical, Educational and Health Psychology, UCL, 1-19 Torrington Place, Camden, London WC1E 7HB, UK; Adapt Lab, Research Department of Clinical, Educational and Health Psychology, UCL, 1-19 Torrington Place, Camden, London WC1E 7HB, UK

**Keywords:** Cardiovascular disease, Depression, Evidence-base psychological therapies, Coronary heart disease, Stroke, Electronic healthcare records, Primary healthcare

## Abstract

**Aims:**

People with depression are up to 72% more at risk to develop cardiovascular disease (CVD) in their lifetime. Evidence-based psychotherapies are first-line interventions for the treatment of depression and are delivered nationally in England through the National Health Service *via* the Improving Access to Psychological Therapy (IAPT) primary care programme. It is currently unknown whether positive therapy outcomes may be associated with cardiovascular risk reduction. This study aimed to examine the association between psychotherapy outcomes for depression and incident CVD.

**Methods and results:**

A cohort of 636 955 individuals who have completed a course of psychotherapy was built from linked electronic healthcare record databases of national coverage in England: the national IAPT database, the Hospital Episode Statistics (HES) database, and the HES–ONS (Office of National Statistics) mortality database. Multivariable Cox models adjusting for clinical and demographic covariates were run to estimate the association between reliable improvement from depression and the risk of subsequent incidence of cardiovascular events. After a median follow-up of 3.1 years, reliable improvement from depression symptoms was associated with a lower risk of new onset of any CVD [hazard ratio (HR): 0.88, 95% confidence interval (CI): 0.86, 0.89], coronary heart disease (HR: 0.89, 95% CI: 0.86, 0.92), stroke (HR: 0.88, 95% CI: 0.83, 0.94), and all-cause mortality (HR: 0.81, 95% CI: 0.78, 0.84). This association was stronger in the under 60 compared with the over 60 for all outcomes. Results were confirmed in sensitivity analyses.

**Conclusion:**

Management of depression through psychological interventions may be associated with reduced risk of CVD. More research is needed to understand the causality of these associations.

## Structured Graphical Abstract

**Structured Graphical Abstract:**
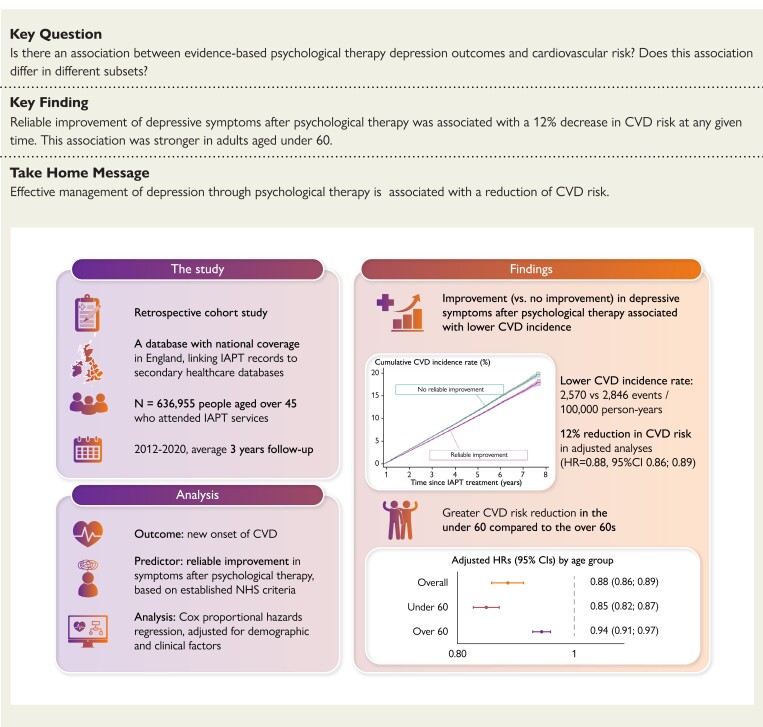
A retrospective cohort of 636 955 individuals was followed up for a median of 3 years after receiving a course of psychological therapy in primary care in England. After adjustment for clinical and demographic factors, those whose depression symptoms improved after therapy were 12% less likely to experience a cardiovascular event than those who did not. Legend: NHS, National Health Service; IAPT, Improving Access to Psychological Therapies, IAPT is delivering psychological therapies for depression across England. CVD, cardiovascular disease; HR, hazard ratio; CI, confidence interval. Cumulative incidence rates were estimated using the Nelson-Aalen method.

**See the editorial comment for this article ‘Successful psychological treatment of depression and subsequent reduction in CVD events’, by D.L. Hare, https://doi.org/10.1093/eurheartj/ehad173.**

## Introduction

Cardiovascular diseases (CVDs) are the leading cause of death, and represent 32% of all deaths worldwide, with 18.6 million people having died from CVD in 2019 globally.^1^ The number of people living with CVD, estimated at 523 million in 2019, continues to increase with the global growth and aging of the population,^1^ and large proportion of the CVD burden is attributable to modifiable risk factors, making CVD prevention a global priority.^2^

The incidence and prevalence of depression is higher among people with CVD than those without CVD^3^ and the risk of CVD is approximately 72% higher among people with major depressive disorders relative to healthy controls.^4^

There are several plausible explanations for the association between earlier depression and later CVD; depression may be a risk factor, or it may be due to reverse directionality whereby having incipient CVD leads to depressive symptoms. If depression were a risk factor, the underpinning mechanisms may be behavioural as health behaviours associated with depression have been shown to increase the risk of CVD. For example, dietary habits, physical activity, tobacco and alcohol consumption^5^ may all improve following depression remission. The mechanisms may also be neurobiological: the vascular consequences of depression-related nervous system and hypothalamic–pituitary–adrenal axis (HPA) dysregulation may be associated with the onset of hypertension and diabetes, both cardiovascular (CV) risk factors, and subsequently the onset of CVD.^6,7^ Evidence also suggests that depression may modulate the genetic risk of CVD.^8^

Given the potential for depression as a risk factor for CVD, a key question is whether effectively treating depression is associated with reduced CVD risk. A recent review examining depression as risk factor for heart disease and stroke identified that most studies to date have focused on evaluating mental health interventions in patients who have already developed CVD.^9^ Those that focussed on people who have not yet developed CVD found that psychotropic medications may exacerbate the risk of CVD onset including for people with major depression.^10^ However, evidence is lacking when it comes to psychological therapies^11^ and the question of whether positive outcomes are associated with a reduction in the risk of CVD onset is unanswered.^9^

This question is of critical clinical importance because evidence-based psychological interventions are a key first-line intervention in the treatment of depression. In the UK, they are recommended by the National Institute for Health and Care Excellence and patients have been found to have a 3-to-1 preference for them compared with anti-depressant medication.^12^ However, to date, no study has investigated whether reducing depression symptoms through psychological therapy is associated with a reduction in the risk of new onset CVD. Denying access to such psychological therapy for people with depression would be both unethical and logistically extremely challenging, so investigating this with randomised controlled trials (RCT) is not possible. Thus, in line with Medical Research Council guidance for evaluating complex interventions in such situations,^13^ this study uses a naturalistic design in a nationally delivered primary care psychological therapy programme; Improving Access to Psychological Therapies (IAPT). IAPT services are freely available across England *via* the National Health Service (NHS) and offer a variety of evidence-based psychological therapies for common mental health problems including depression.

The primary aim of this study was to evaluate the association between psychotherapy treatment outcome and risk of incident all-cause CVD, its most common subtypes within the UK^14^ [coronary heart disease (CHD) and stroke], as well as all-cause mortality. The secondary aim was to investigate whether this association may be stronger in specific subgroups of people, based on most common CV risk factors routinely collected in NHS services and depression treatment prognostic factors (age, gender, ethnicity, economic deprivation, long-term health conditions, hypertension, diabetes, anti-depressant medication, reason for treatment discontinuation).^14,15^ Finally, a *post-hoc* aim was to evaluate plausibility of the presence of a bi-directional association.

## Methods

### Study design

A retrospective observational cohort study design was used to assess the association between outcomes of evidence-based psychotherapies for depression and new incidence of CV events, using NHS Digital-linked electronic healthcare records databases from England. The databases included were the IAPT national database, the Hospital Episode Statistics (HES) database, and its linked Office of National Statistics (ONS) mortality database (the HES–ONS dataset). Each database covers all services in all healthcare regions across the country.

This study followed the Enhancing the Quality and Transparency of Health Research (EQUATOR) reporting guidelines: REporting of studies Conducted using Observational Routinely-collected health Data (RECORD).^16^

### Data sources

All three data sources were fully anonymised, and a linkage key was provided by NHS Digital, for records from each database to be linked at the individual patient level using an anonymised subject identifier. Non-identifiable information was provided by NHS Digital with a legal basis for the anonymization, meaning this research did not require research ethics committee review, as per the Governance Arrangements of Research Ethics Committees (GAfREC).

#### The IAPT database

In England, IAPT services are freely available *via* the NHS and offer a range of evidence-based psychological therapies for common mental health problems, including depression. The IAPT programme started in 2008, and national statistics data collection started in 2012.^17^ The IAPT service was generally found to be cost-efficient,^18^ and receiving IAPT psychotherapy is also associated with reduction in hospital utilization.^19^

IAPT services provide evidence-based psychological treatments (e.g. cognitive behavioural therapy, interpersonal therapy) for anxiety and depression in one-to-one or group settings, following a stepped-care model, in which intensity of interventions is adapted according to people’s needs following national recommendations^20^ (see Supplementary data online, *Supplement A* for more details).

Over 500 000 individuals complete a course of treatment every year.^21^ One course of treatment may include both low and high intensity therapy. The end of a course of treatment is defined by IAPT clinicians and is based on whether individuals have achieved improvement criteria, need to be referred to secondary care services, or decide to withdraw from treatment.

The IAPT database includes all people who accessed IAPT services across England between 2012 and 2019. Data are organised by course of treatment, and routinely collected by all IAPT services following national information standards.^22^

#### The HES database

The HES database includes routinely collected details of admissions, outpatient appointments, and accident and emergency (A&E) attendances at NHS hospitals in England.^23^ Hospital discharge diagnoses are collected using the International Classification of Diseases 10th revision (ICD-10) diagnosis codes. At the time of analysis, complete HES data were available to 31 March 2020. It is challenging to accurately diagnose CVD in an A&E context^24^ with diagnosis codes reflecting presenting problems rather than clinical diagnoses. For this reason, records from the HES A&E dataset were not included.

#### HES–ONS mortality data

The HES–ONS mortality dataset is a linkage of the HES database with death certificates held by the ONS. This dataset provides the date and cause of death of people treated in English hospitals, whether they died in hospital or not. At the time of analysis, HES–ONS mortality data were available to 1 June 2020.^25^

### Study period and study population

A retrospective cohort was formed of those who have completed a course of treatment (at least two IAPT treatment sessions as per previous research and NHS Digital guidelines^26,27^). When individuals had received several courses of IAPT treatment between 2012 and 2019, the first course of treatment was considered as per previous research^28^ to maximise the length of the study follow-up. As prevalence of CVD rises between the age of 45 and 54,^29^ individuals were included in the analytic cohort if they were over 45 years of age at the time of IAPT referral, had completed depression symptom measures pre- and post-treatment, and had at least one linked HES or ONS record available. As dementia may also be associated with CVD and lead to different therapy outcomes,^30,31^ individuals were excluded if they had a dementia diagnosis code prior to completion of psychological treatment (as defined in *Table 1*).^32–40^

**Table 1:** Available data and measures

Data item	Data source	Information on measurement
**Outcome measures—cardiovascular disease ascertainment**		
CV	HES	Date of incident CV event was defined as the first occurrence of any of the following ICD-10 codes: I20–25, I26–I28, I30–I52, I60–I69, and I70–79^32^
CHD	HES	Date of incident CHD was assessed as the first occurrence of any of the following ICD-10 codes: I20–I25^32^
Stroke	HES	Date of incident stroke was assessed as the first occurrence of any of the following ICD-10 codes: I60, I61, I63, I64^33^
Mortality	HES–ONS	Mortality status was obtained using the date of death captured in the HES–ONS dataset, regardless of whether the death occurred on the same day as a CV event
**Predictors of interest**		
Depression symptoms	IAPT	Self-reported using the Patient Health Questionnaire 9-item (PHQ-9)^34^
		To measure symptoms of depression, scores of 10 or above indicate clinical caseness for depression, and a reduction of 6 or more points was used to indicate reliable decrease in symptoms^21^
**Covariates**		
Demographic covariates	IAPT	At the point of referral: self-reported gender, age, Index of Multiple Deprivation (IMD) quintile (a lowest IMD indicates a higher deprivation area), and ethnicity (based on UK census codes ‘White’, ‘Mixed’, ‘Asian’, ‘Black’, ‘Chinese’, and ‘other’) were available in the dataset
**Clinical and biological covariates**		
Anxiety symptoms	IAPT	Self-reported using the Generalized Anxiety Disorder Scale 7-item version (GAD-7)^35^
		To assess generalised anxiety symptoms, a cut-off of 8 or higher was used for caseness. An increase of 4 or more points was used to indicate a reliable deterioration in symptoms.^21^ Anxiety measures were used as covariates in the analyses and are also included in definitions of reliable improvement and recovery from depression
Long-term health conditions	IAPT	All patients are asked whether they have any long-term physical health condition (LTC) at referral. The type of condition was not available in the dataset. Presence of a long-term condition may be associated with an adaptation in the therapy provided^36^
Hypertension	HES	Hypertension before the end of IAPT treatment, defined as the presence of an hypertension diagnosis code (yes/no) (ICD-10 codes I10–I11, as in Walker *et al*.^37^)
Diabetes	HES	Diabetes before the end of IAPT treatment, defined as the presence of a diabetes diagnosis code (yes/no) (ICD-10 codes: E10–14, as in Rafnsson and Bhopal^38^)
Psychotropic medication used before treatment	IAPT	Clinicians in the services routinely record whether their patients were prescribed psychotropic medication(s) before treatment
Treatment factors	IAPT	The number of treatment sessions received, reason for treatment discontinuation (completed, dropout, not suitable, declined, referred to another service), year of referral
**Other health indicators**		
Dementia	HES	Individuals with a dementia diagnosis code prior to the end of IAPT treatment (ICD-10 codes: E512, F00, F01, F02, F03, F10.6, F10.7, G30, G31.0)^39^ were excluded

CHD, coronary heart disease; CV, cardiovascular; HES, Hospital Episode Statistics; ICD-10, International Classification of Diseases 10th revision; IAPT, Improving Access to Psychological Therapies; LTC, Long-Term Condition; ONS, Office of National Statistics.

Adapted with permission from Buckman *et al*.^40^

To account for possible bidirectionality of the association between depression and CVDs, the observation period used for analysis started one year (365 days) after the end of IAPT treatment. This was to mitigate any impact of subclinical CV conditions that may have been present before or during IAPT treatment. As in previous studies,^41^ the study period ended on the earliest of the observed date of death and the last available date when HES records could be considered complete (31 March 2020).

Individuals were excluded if they had experienced a CV event before the index date or had <365 days of follow-up between the end of IAPT treatment and the end of the study period, or if they did not meet ‘caseness’, i.e. a clinical threshold criterion for depression (described below). Exclusion criteria are defined in *Table 1*.

**Figure 1:**
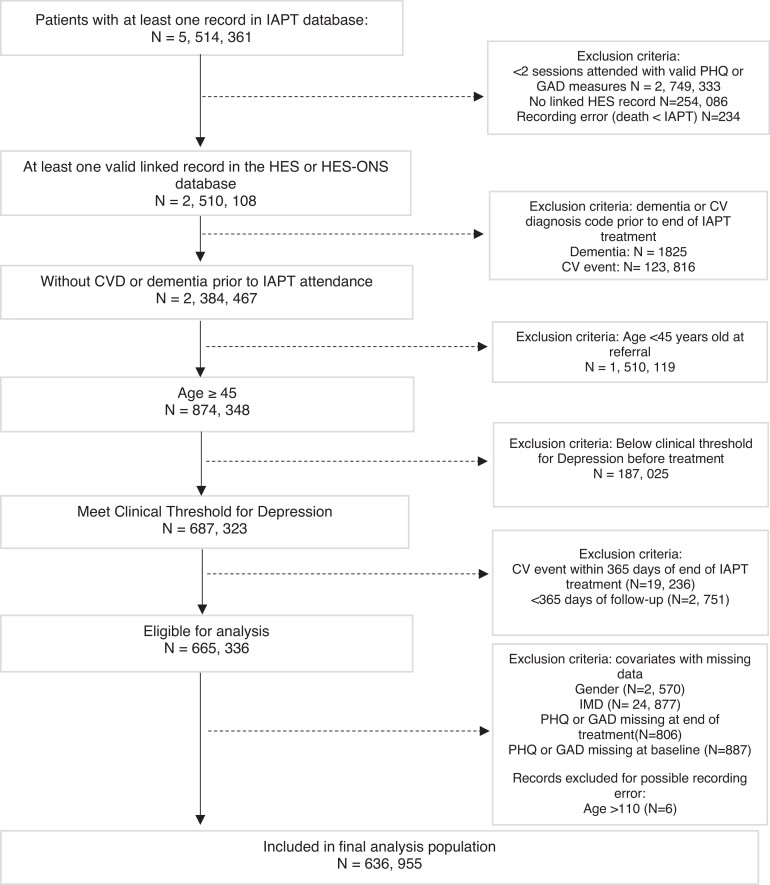
Study flow chart. IAPT, Improving Access to Psychological Therapies; HES, Hospital Episode Statistics; CV, cardiovascular; IMD, Index of Multiple Deprivation.

After applying these inclusion and exclusion criteria, 636 955 individuals were retained in the study cohort. A flow chart is shown in *Figure 1*.

### Measures

A summary of data and measures along with a summary of CVD ascertainment is available in *Table 1*.

#### Outcomes

HES data have previously been found to have good validity in identifying CV outcomes.^33^ ICD-10 codes were used to identify CV events as in previous research (*Table 1*). A new event was defined as the first occurrence of a diagnosis code in any of the hospital discharge diagnosis fields. Mortality status was obtained using date of death captured in the HES–ONS dataset. Time to each type of incident CV event was measured in years from the index date (365 days after IAPT treatment) until the first observed event.

#### Primary predictor: reliable improvement from depression

In line with improvement measures defined by IAPT services, reliable improvement from depression was the primary predictor of interest in the analyses.^26,42^ These metrics were calculated by NHS services according to reliable change criteria which capture the degree of change in symptoms beyond which we can be confident that the change was not due to chance alone, and are routinely used to evaluate IAPT outcomes.^26,43^ In line with IAPT definitions reliable improvement in depression symptoms was (i) a reliable improvement (≥6 points) in depressive symptoms [as measured by the Patient Health Questionnaire 9-item (PHQ-9) score] and (ii) no reliable deterioration in anxiety [≥4 points, as measured by the Generalized Anxiety Disorder Scale 7-item (GAD-7)]) symptoms, between the start and end of treatment.^26^ This criterion is based on both depressive and anxiety symptoms, so that therapy outcome cannot be considered to be good (as above) if a reliable improvement in depression is experienced with simultaneous worsening of anxiety symptoms.

To understand the sensitivity of the association to various IAPT outcomes, two additional predictors of interest were used sequentially in separate statistical models in further analyses:

(1) ‘Reliable recovery’ was achieved when the reliable improvement threshold was met on the PHQ-9, and neither the PHQ-9 nor GAD-7 scores were above clinical cut-offs for caseness at the end of treatment, as defined in *Table 1* and in the IAPT manual.^26^
(2) Change scores in depression symptoms over the course of treatment, derived by the difference between the total PHQ-9 observed before and at the end of treatment, were also used as a measure of improvement.

#### Covariates

Socio-demographic factors such as age, ethnicity, gender, and socioeconomic deprivation account for a large proportion of the variance in CV risk,^44–46^ and have also been linked with depression treatment outcomes.^47–52^ These risk factors of CVD along other clinical treatment factors identified in the literature (diabetes, hypertension, long-term health conditions^19^) as well as treatment factors (such as reason for stopping treatment and number of sessions^53^) were included as covariates in the analyses, where they were available in the databases, and are described in *Table 1*.

### Data analysis

Unless otherwise stated, all analyses were pre-specified and conducted using STATA 16.0. Analyses were developed to not rely solely on null hypothesis significance testing.^54^ As such, results were interpreted considering both their statistical and clinical relevance.

#### Descriptive analyses

Baseline and demographic characteristics at the start of IAPT treatment were summarised using descriptive statistics.

Nelson-Aalen cumulative hazard plots were used to estimate the cumulative incidence rate of each outcome. Absolute risk and absolute risk differences estimates were obtained at the average median follow-up time (4 years after the end of IAPT treatment).

#### Missing data

For categorical covariates, when more than 5% of individuals had missing values for a covariate, a ‘missing’ category was included in analyses, and results were compared with the reference category to understand the impact of data missingness.^49^

Missing data for other covariates accounted for <5% of the sample. These records were excluded since previous research has found the impact of missing data may be negligible in such cases.^55^

#### Primary statistical analyses

##### Reliable improvement in depression symptoms and incident cardiovascular events

Multivariable Cox proportional hazards models were used to test associations between reliable improvement from depression following IAPT treatment, and time to incident CV events. For people who did not experience a CV event, results were censored at the earliest between the date of death, or the end of the study period (31 March 2020).^41^ Analyses were conducted for each of all-cause CV events, CHD, stroke, and all-cause mortality.

To evaluate the confounding effects of different clinical and socio-demographic factors that may be associated with both incident CVD and depression treatment outcomes, Cox regressions of time to first incidence of a CV event were fitted, including reliable improvement as the main predictor, adjusting for additional covariates in the following sequence: Model 1: no additional covariates; Model 2: demographic covariates [age, gender, ethnicity, Index of Multiple Deprivation (IMD)]; Model 3: Model 2 + clinical covariates (CV risk factors and other treatment factors). Model 3 was considered the primary model.

The Breslow method was used to account for ties in the data.^56^ For each covariate of interest, hazard ratios (HRs) and their 95% confidence intervals (CIs) were calculated using the Wald approximation.

The proportional hazard assumption was checked using statistical and visual tests, for each of the variables individually, and for the full model, using Schoenfeld residuals tests.^57^ When the assumption of proportional hazards could not be sustained for individual covariates, baseline hazards were stratified by each level of these covariates.

To assess the linearity of the association between the outcome and continuous covariates, both categorical and continuous covariates were fitted in the models sequentially for the continuous measures (age, IMD rank, baseline PHQ-9 score, and baseline GAD-7 score). The categorised version was retained in the final model if it provided a better fit to the data. When available, previously validated clinical cut-offs were used. This method allowed a pragmatic interpretation of the results and has previously been found acceptable when analyses are sufficiently powered.^58,59^

##### Sensitivity analyses

Sensitivity analyses were conducted using the same method as for primary analyses, using reliable recovery and change in symptom scores as main predictors of interest. To evaluate the robustness of the results, analyses were also repeated using another definition for the index date, starting two years (730 days) after the end treatment. Analyses were also re-run using the last course of treatment received in IAPT instead of the first and additionally adjusting for repeat attendance.

To understand the effect of the competing risk of death, the analyses were re-run considering death as an event, and CV events as censored observations.^60^

##### Post-hoc analyses: reverse directionality

In the presence of a bi-directional association, there is a possibility that a group of people do not improve in their depression symptoms after therapy due to incipient CVD. If this was the case, it would be expected that people who do not reliably improve would be at increased risk of experiencing a CV event than those who do in the first months after therapy. This would in turn lead to larger HRs immediately after the end of therapy. To evaluate how HRs vary over time, we fitted flexible parametric survival models to the incidence of all CV events, adjusting for all available covariates (as in Model 3). Models were fitted on the log-hazard scale to account for the non-proportionality of hazards for multiple covariates.^61^ A restricted cubic spline with five degrees of freedom was used for the baseline hazard, knots were placed at the quintiles of the event times. Likelihood ratio tests were used to test the proportional hazard assumption, by adding time by covariate interaction terms for each of the covariates. When the proportionality of hazards was not met, time-dependent effects were accounted for by adding time by covariate interaction terms to the model, using a spline with one degree of freedom.

#### Secondary analyses: interactions and differential effects

Tests for an interaction between each demographic or clinical covariates and the reliable improvement indicator were carried out by adding an interaction term to the fully adjusted ‘all CV’ model sequentially for each covariate and conducting a Wald interaction test. When a global interaction was found for a covariate, the presence of differential effects was further evaluated for each level of the covariates, by conducting Wald tests for simple or composite linear combination of the regression parameters.

##### Post-hoc analysis stratified by age

Interaction analyses revealed the magnitude of the associations differed according to two broader age categories (<60 or ≥60 years old). To further explore these age differences, analyses were re-run separately for these two age strata for all outcomes, following the same methods as for the primary analyses.

## Results

### Descriptive analyses

Demographic and baseline characteristics are presented in *Table 2*. The study cohort included a higher proportion of women (65.6%), with an average age of 55 (SD = 8.06).

**Table 2:** Demographic and baseline characteristics

Baseline characteristic	Study cohort (*N* = 636 955)	With reliable improvement (*N* = 373 623)	Without reliable improvement (*N* = 263 332)
**Age, years**			
ȃMean (SD)	55.0 (8.06)	55.4 (8.22)	54.5 (7.8)
ȃRange	45–101	45–101	45–101
**Age, years (category, %)**			
ȃ45–49	29.2	28.0	30.9
ȃ50–54	26.4	25.6	27.2
ȃ55–60	19.6	19.5	19.8
ȃ60–64	11.8	12.3	11.0
ȃ65–69	6.7	7.5	5.7
ȃ70–74	3.6	4.0	2.9
ȃ75–79	1.8	1.9	1.5
ȃ80–84	0.7	0.8	0.7
ȃ ≥ 85	0.3	0.3	0.3
**Gender (%)**			
ȃMale	34.4	33.4	35.1
ȃFemale	65.6	66.1	64.9
**Ethnicity (%)**			
ȃWhite	83.5	85.0	81.1
ȃMixed	1.1	1.0	1.3
ȃAsian	3.0	2.6	3.6
ȃBlack	2.4	2.3	2.7
ȃChinese	0.1	0.1	0.1
ȃOther	1.0	0.8	1.2
ȃMissing	8.9	8.3	9.9
**IMD quintiles (%)**			
ȃ1st	21.0	18.5	24.6
ȃ2nd	20.8	20.1	21.8
ȃ3rd	20.5	21.0	19.8
ȃ4th	19.5	20.1	18.0
ȃ5th	18.2	19.8	15.9
**PHQ-9 (Mean, SD)**			
ȃBefore treatment	17.5 (4.6)	17.8 (4.4)	17.1 (4.8)
ȃEnd of treatment	10.2 (7.1)	5.6 (4.3)	16.3 (5.7)
ȃChange (decrease)	7.2 (6.7)	11.7 (4.3)	1.1 (4.2)
**GAD-7 (Mean, SD)**			
ȃBefore treatment	14.6 (4.6)	14.6 (4.5)	13.9 (4.7)
ȃEnd of treatment	8.8 (6.1)	5.5 (4.3)	7.7 (5.8)
ȃChange (decrease)	5.7 (6.1)	9.1 (4.9)	6.2 (6.0)
Reliable improvement (%)	58.7	100	0
Reliable recovery (%)	43.0	73.2	0
**Psychotropic medication (%)**			
ȃYes	54.7	54.3	55.2
ȃNo	35.0	36.1	33.3
ȃMissing	10.3	9.5	11.5
Diabetes before treatment (%)	4.2	3.9	4.5
Hypertension before treatment (%)	10.1	10.1	10.1
**Long-term health condition (%)**			
ȃYes	30.8	29.5	32.7
ȃNo	46.2	48.9	42.5
ȃMissing	22.9	21.7	24.8
**Reason for treatment end (%)**			
ȃCompleted	46.7	58.9	29.2
ȃDropout	15.9	10.7	23.2
ȃNot suitable	0.9	0.4	1.8
ȃDeclined	2.4	1.4	3.8
ȃReferred to another service	3.1	1.4	5.6
ȃMissing	31.1	27.2	36.7
Number of sessions (Mean, SD)	6.7 (4.7)	7.3 (4.7)	5.7 (4.6)
Time between start and end of therapy (weeks) (Mean, SD)	17.7 (15.0)	19.0 (14.7)	15.8 (15.2)

GAD-7, Generalized Anxiety Disorder Scale 7-item; IMD, Index of Multiple Deprivation; PHQ-9, Patient Health Questionnaire 9-item; SD, standard deviation.

The median follow-up time after the index date was 3.1 years (range 0.0–7.0 years); 1.3% of individuals died before experiencing a CV event and were censored in the analyses and 7.8% individuals experienced an incident CV event, with an incidence rate of 2687 per 100 000 person-years.

**Figure 2:**
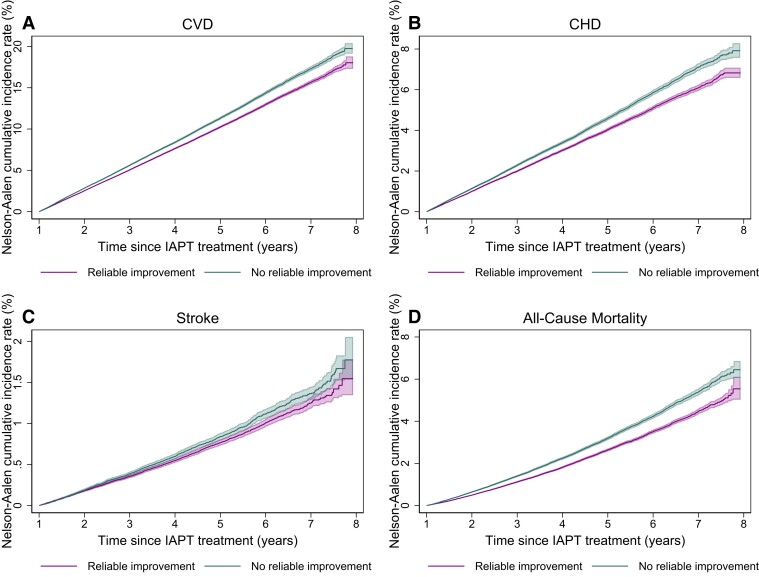
Nelson-Aalen curve of cumulative incidence of CVD (*A*), CHD (*B*), stroke (*C*), and all-cause mortality (*D*), by improvement status after therapy. CVD, cardiovascular disease; CHD, coronary heart disease; IAPT, Improving Access to Psychological Therapies.

Nelson-Aalen cumulative incidence estimates along with 95% CIs are presented in *Figure 2*.

Unadjusted absolute risk at 4 years and incidence rates of CV events and all-cause mortality were lower for people with reliable symptom improvement after psychological treatment (*Table 3*). Four years after IAPT, the absolute risk of experiencing a CV event was 7.64% for people with reliable improvement, and 8.38% for people without, an absolute risk difference of 0.74%.

**Table 3:** Cardiovascular events incidence, with or without reliable improvement from depression

Outcome	Overall			With reliable improvement			Without reliable improvement		
	(*N* = 636 955)			(*N* = 373 623)			(*N* = 263 332)		
	*n* events	IR/100 000 PY	Absolute risk at 4 years (%)	*n* events	IR/100 000 PY	Absolute risk at 4 years (%)	*n* events	IR/100 000 PY	Absolute risk at 4 years (%)
**All CV events**	49 803	2687	7.95	27 526	2570	7.64	22 277	2846	8.38
ȃCHD	20 470	1073	3.18	11 136	1012	3.02	9334	1156	3.39
ȃStroke	3943	203	0.57	2173	194	0.55	1770	215	0.60
Death from any cause	14 125	724	2.00	7392	658	1.81	6733	814	2.24

Absolute risk was calculated from the Nelson-Aalen estimator, 4 years after the end of IAPT treatment.

IR, incidence rate; *n*, number of incident events; PY, person-years.

### Primary statistical analyses

Results of the primary analyses are presented in *Table 4* and suggest an association between reliable improvement from depression and a decreased risk of incident all-cause CV events, CHD, and stroke, regardless of adjustment for demographic and/or clinical covariates.

**Table 4:** Adjusted hazard ratios for cardiovascular outcomes associated with reliable improvement from depression

Study cohort (*N* = 636 955) covariate	Model 1	*P*-value	Model 2	*P*-value	Model 3	*P*-value
	HR (95% CI)		HR (95% CI)		HR (95% CI)	
All CV	0.90 (0.89;0.92)	<0.001	0.87 (0.85;0.88)	<0.001	0.88 (0.86;0.89)	<0.001
CHD	0.88 (0.85, 0.90)	<0.001	0.86 (0.84; 0.89)^a^	<0.001	0.89 (0.86; 0.92)^b^	<0.001
Stroke	0.91 (0.85, 0.97)	<0.001	0.87 (0.85; 0.92)	<0.001	0.88 (0.83; 0.94)	<0.001
Death from any cause	0.82 (0.79, 0.85)	<0.001	0.77 (0.74; 0.79)^c^	<0.001	0.81 (0.78; 0.84)^c^	<0.001

Model 1: reliable improvement from depression indicator (yes vs. no).

Model 2: Model 1 + demographic covariates (age, gender, ethnicity, IMD quintile).

Model 3: Model 2 + clinical covariates (baseline PHQ-9 and GAD-7 scores, psychotropic medications, long-term health condition, diabetes or hypertension at baseline, reason for ending treatment, year of appointment, number of sessions).

Age, Baseline PHQ score, IMD rank were fitted as categorical variables.

CHD, coronary heart disease; CI, confidence interval; CV, cardiovascular; HR, hazard ratio.

Baseline hazard stratified by age group.

Baseline hazard stratified by age group and PHQ category.

Baseline hazard stratified by age group and IMD quintile.

An HR of 0.88 [95% CI (0.86, 0.89)] was observed after adjusting for demographic and clinical covariates, indicating that at any given time, the risk of experiencing any incident CV event was reduced by 12% for individuals who reliably improved after psychological therapy, compared with those who did not. The magnitude of the HRs was similar in all models, with the models including demographic factors only yielding slightly lower HRs than the univariable model and the fully adjusted model, and higher effect sizes observed in the mortality models. This suggests that reliable improvement from depression was associated with a lower incidence of CV events and mortality, independently of clinical and demographic factors.

The proportional hazard assumption was met in the CV and stroke models for all covariates. In the CHD and mortality models, the assumption was met after baseline hazards were stratified by age group, PHQ-9 category, and/or IMD quintile (see *Table 4* for more details).

#### Sensitivity analyses

The associations observed in the primary model were replicated when including reliable recovery or change in symptom scores as main predictors, instead of reliable improvement (see Supplementary data online, *Supplement B*).

Analyses starting the observation period 2 years after psychological treatment revealed no difference in the results, suggesting good statistical robustness of the analyses (see Supplementary data online, *Supplement C*).

Similarly, using the last course of treatment instead of the first in the analysis did not yield different results (see Supplementary data online, *Supplement D*).

Sensitivity analyses considering the competing risk of death before CV event showed that reliable improvement was not associated with an increased risk of dying from other causes, which suggests that the competing risk of death did not impact the results.

#### Post-hoc analyses: reverse directionality

**Figure 3:**
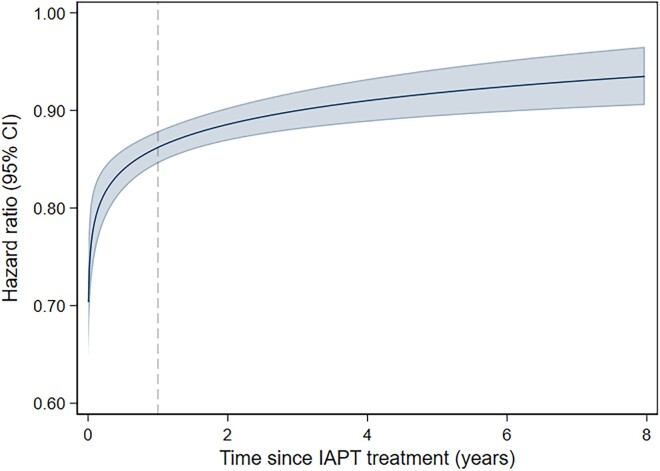
Adjusted hazard ratios for CVD associated with reliable improvement from depression (vs. no improvement from depression). Note: Models includes reliable improvement from depression indicator (yes vs. no), demographic covariates (age, gender, ethnicity, IMD quintile) and clinical covariates (baseline PHQ-9 and GAD-7 scores, psychotropic medications, long-term health condition, diabetes or hypertension at baseline, reason for ending treatment, year of appointment, number of sessions). Age, Baseline PHQ score, IMD rank were fitted as categorical variables. Model includes time-dependent covariates for reliable improvement, age category, psychotropic mediations, long-term health condition, year of appointment, and reason for ending treatment.

After adjustment for covariates, flexible parametric survival models revealed a sharp increase in the HR in the first year after IAPT treatment (*Figure 3*), suggesting that people whose depression symptoms do not improve after therapy are at heightened risk of experiencing a CV event in the first year after therapy. Then, the HR progressively reached a similar magnitude as for the main analyses, with a fully adjusted HR of 0.89 (95% CI 0.88, 0.92) 4 years after IAPT.

### Secondary analyses: interactions and differential effects

Interaction tests between the reliable improvement indicator and each individual covariate in the main CV model are presented in Supplementary data online, *Supplement E*. These tests only revealed an interaction between age category and the reliable improvement indicator [χ^2^ (8, *N* = 636 955) = 36.33, *P* < 0.001]. Further statistical tests revealed that this interaction was especially apparent in age categories under 60 years old, compared with the over 60 categories (see Supplementary data online, *Supplement E* for more details).

To further characterise potential differential effects for people <60 compared with the over 60s, two age strata were created *post-hoc*, and models were re-run separately for both strata across all outcomes (see Supplementary data online, *Supplement F* for demographic characteristics). HRs associated with each outcome for the reliable improvement variable in the fully adjusted models are presented by stratum in *Table 5*.

**Table 5:** Incidence and risk of CV events with or without reliable improvement from depression, by age stratum

Outcome	Age < 60 (*N* = 478 864)			Age ≥ 60 (*N* = 158 091)		
	With/without reliable improvement	Fully adjusted model HR (95% CI)	*P*-value	With/without reliable improvement	Fully adjusted model HR (95% CI)	*P*-value
	unadjusted IR/100 000 PY			unadjusted IR/100 000 PY		
All CV	1745/1939	0.85 (0.82; 0.87)	<0.001	5091/5509	0.94 (0.91; 0.97)	<0.001^a^
CHD	921/724	0.86 (0.83; 0.90)	<0.001	1860/2074	0.94 (0.90; 0.99)	0.009^b^
Stroke	162/127	0.85 (0.78; 0.93)	0.001	387/417	0.93 (0.85; 1.03)	0.179
Death from any cause	552/380	0.77 (0.73; 0.81)	<0.001^c^	1460/1813	0.85 (0.80; 0.89)	<0.001^d^

Model 3: reliable improvement (yes vs. no) + demographic covariates (age category, gender, ethnicity, IMD quintile) + clinical covariates (baseline PHQ-9 and GAD-7 scores, psychotropic medications, long-term health condition, diabetes or hypertension at baseline, reason for ending treatment, year of appointment, number of sessions).

Separate models were fitted for each stratum.

Age, Baseline PHQ score, IMD rank were fitted as categorical variables.

Interpretation: For All CV, an HR of 0.85 in the under 60 corresponds to a 15% decrease in incident risk at any given time (6% for the over 60).

For CHD, an HR of 0.86 in the under 60 category corresponds to a 14% decrease in incident risk at any given time (6% for the over 60).

For stroke, an HR of 0.85 in the under 60 category corresponds to a 15% decrease in incident risk at any given time (7% for the over 60).

For all-cause mortality, an HR of 0.77 in the under 60 category corresponds to a 23% decrease in incident risk at any given time (15% for the over 60).

CHD, coronary heart disease; CI, confidence interval; CV, cardiovascular; HR, hazard ratio; IR, incidence rate; PY, person-years.

Baseline hazard stratified by age category and presence of long-term health conditions.

Baseline hazard stratified by age category and PHQ category.

Baseline hazard stratified by IMQ quintile.

Baseline hazard stratified by age category.

The proportional hazard assumption was met for all covariates after stratification of baseline hazard according to age category, presence of long-term health conditions, PHQ-9 category, and/or IMD quintile (see *Table 5* for more details).

The decrease in risk of incident CVD in association with reliable improvement from psychological treatment was higher in individuals below 60 years of age, an effect which was observed regardless of the type of CV event. This difference was also observed in both strata for mortality outcomes, in which there was also a greater decrease in risk in comparison to CV events. As for the main analyses, we did not find evidence that results were influenced by the competing risk of death in either age stratum.

## Discussion

We found that people whose depression symptoms reliably improved after psychological treatment experienced fewer incident CV events over an average of 3 years of follow-up, compared with those who did not. After adjustment for demographic and clinical covariates, reliable improvement from depression was associated with a 12% decrease in incident risk of CVD at any given time, with similar results observed for CHD, stroke, and all-cause mortality. The association was stronger in people below 60 years old than people aged 60 and over (15% vs. 6% decreased risk of incident CVD and 22% vs. 15% decreased risk of all-cause mortality, respectively) (*Table 5* and Structured Graphical Abstract). These findings are important as they suggest that successful outcomes of evidence-based psychological interventions may extend beyond psychological health and have long-term physical health benefits, particularly for those aged under 60. Sensitivity analyses indicated the associations were statistically robust to therapy outcomes definitions and were replicated when starting follow-up 2 years after the end of psychological therapy.

In *post-hoc* analyses of reverse directionality, a sharp increase in the HR was observed in the first year after treatment, before reaching a similar magnitude as for the main analyses. This observation in the first year after treatment is in line with the idea that there is an initial period where reverse causality may have an influence. Specifically, preclinical CV symptoms during therapy may lead to poorer depression outcomes and be associated with a higher CV risk immediately after therapy.

This study extends previous literature by evaluating the association between psychological treatment outcomes and incident CVD and is the first to examine psychological therapies as a CV risk modifier in a large sample with national coverage. The magnitude of the overall association, with a decrease in risk of CVD of 10%–15% after one round of psychological treatment, is comparable to the effect observed in studies investigating the utility of low lipid or carbohydrate diets^62^ and collaborative care interventions^63^ in CV risk modification.

The reduced magnitude of association in older adults is consistent with previous research, in that CV risk reduction interventions in general may be less effective in older adults,^64^ highlighting the importance of early intervention for people at risk of CVD. Our findings suggest that these differences cannot be explained by the competing risk of death. These differences are perhaps explained by the fact that older adults are more likely to experience physical frailty, which has previously been associated with poorer CV outcomes, but also with poorer outcomes for CV risk factor modification interventions, such as blood pressure reduction or glycaemic control.^65^ It has also been found that lifestyle interventions may reduce the risk of developing frailty, but not in reversing frailty when it is already present.^66^ It is possible that due to accumulated health problems in frail older adults, the mediating effect of lifestyle changes after successful psychological treatment is less strong in this population.

We did not evaluate mechanisms underpinning the observed associations. However, since lifestyle interventions have been found to be effective in reducing incident CVD, it is plausible that successful psychological treatment may facilitate lifestyle changes that are protective of CVD.^67,68^ Similarly, some, albeit mixed evidence suggests that psychological therapy may influence biological markers of CV risk such as blood pressure, and inflammatory biomarkers,^69^ or through HPA axis regulation.^70,71^ Age-related elevation of inflammatory biomarkers may also explain the reduced magnitude of the association for older adults.^72^

### Strengths and limitations

A strength of this study is the use of a big data resource including data from all IAPT services in England, comprising a very large sample size with national coverage, providing generalisability of these findings to the population who accesses those services. Moreover, comparisons with effect sizes reported in previous research suggest that the effect sizes identified are clinically meaningful.

This study has some limitations, including those common to observational studies. The relationships reported here are only longitudinal associations between successful therapy outcomes and decreased risk of CV onset. Results cannot tell us whether the reduction of CV risk is caused by the reduction of depression symptoms, and it is not known whether individuals at risk of CVD may have additionally received lifestyle interventions. One potential explanation for these results is reverse causality, whereby undiagnosed CVD or CVD risk inducing lifestyle (smoking/lack of activity) may lead to poorer treatment outcomes. An RCT would be needed to understand whether these associations reflect a causal pathway and to confirm the differential effect of age. However, it may not be possible to conduct such an RCT due to logistical and ethical reasons detailed previously. Moreover, due to lack of data availability, external validity could not be assessed to establish the generalisability of those results beyond the population of people with depression who are seeking treatment. This study was conducted over a median follow-up of 3 years, meaning that more research is needed to evaluate the magnitude of the associations in the longer term as CVD is a slowly developing disease.

Finally, data were not available for some potential confounders identified in the literature. No data were available on obesity,^73^ physical activity,^74,75^ social support,^40,76^ alcohol, or tobacco use.^77^ These variables are thought to be associated with depression treatment outcomes and maybe instrumental in the modification of CV risk^78^ suggesting that future research is called for to understand their role in these associations.

### Research and clinical implications

Future research may focus on evaluating the causality of the association, by making observational work well aligned with criteria for approximating causality^79^ as well as increasing sample representativeness, including groups currently underrepresented in mental health services. Obtaining a comprehensive picture of CV risk at baseline, including biological and behavioural or lifestyle measures, will allow researchers to understand whether psychological treatment outcomes may differ according to the intensity of baseline CV risk, health behaviours and age, and allow for more robust evaluation of reverse directionality. Our results also suggest that response to psychological interventions could potentially inform future CV risk stratification and management strategies. Further work could examine this in the context of more established biological and behavioural risk factors. Understanding the mediating role of lifestyle changes in the relationship between improvement from symptoms of depression and CVD would allow further development of tailored interventions for people at risk of CVD, by targeting realistic treatment goals that may be protective of CVD.

Findings from this study suggest that management of depression through psychological interventions could be instrumental in preventing CVD. IAPT offers opportunity to access psychological therapies to over a million adults a year in England, and such programmes are currently not widespread in Europe and across the world. Findings from this study suggests that increasing access to evidence-based psychological therapies in line with patient preferences and clinical guidelines could extend beyond mental health outcomes, have long-term physical health benefits, and further reduce both the clinical and economic burden of CVDs. This is especially relevant for groups currently underrepresented in mental health services, such as people over 65^27^ and people from minoritised ethnic groups, who have expressed perceiving more barriers to accessing those services^80,81^ and in some cases may be at greater risk of CV events.

Finally, integrated care models of mental and physical health have been successful for people with long-term physical health conditions,^36^ and extending those models for people at higher risk of CVD could be one way to increase access to services to underrepresented populations.^82^

## Supplementary Material

ehad188_Supplementary_Data

## Data Availability

All data used for this study are available upon successful application to NHS Digital *via* the Data Access Request Service (DARS): https://digital.nhs.uk/services/data-access-request-service-dars. Data fields can be accessed *via* NHS Digital data dictionary: https://www.datadictionary.nhs.uk/.
