# Supplementary material for: Psychological therapies for depression and cardiovascular risk: evidence from national healthcare records in England
Source: Eur Heart J. 2023 Apr 18;44(18):1650–62. doi: 10.1093/eurheartj/ehad188 (PMC10163979; doi:10.1093/eurheartj/ehad188)
Supplement: ehad188_Supplementary_Data [file ehad188_supplementary_data.docx]

**Psychological therapies for depression and cardiovascular risk: evidence from national healthcare records in England**

Supplementary files

Table of contents

[Supplement A – NICE-recommended psychological interventions provided in IAPT 3](#_Toc121911668)

[Supplement B - Sensitivity analyses - Adjusted hazard ratios for cardiovascular outcomes associated with reliable recovery from depression and change in depressive symptoms scores 4](#_Toc121911669)

[Supplement C - Sensitivity analyses - Adjusted hazard ratios for cardiovascular outcomes associated with therapy depression outcomes starting the observation period two years after end of treatment 5](#_Toc121911670)

[Supplement D - Sensitivity analyses - Adjusted hazard ratios for cardiovascular outcomes associated with therapy depression outcomes, measuring depression symptoms improvement at the last course of IAPT treatment 6](#_Toc121911671)

[Supplement E - Interaction tests and adjusted Hazard Ratios by age stratum in All CV events fully adjusted model 7](#_Toc121911672)

[Supplement F - Demographic and baseline characteristics by age stratum 9](#_Toc121911673)

Supplement A – NICE-recommended psychological interventions provided in IAPT

People with less complex problems are commonly offered short-term (<=8 sessions) “low-intensity” guided self-help therapies, which can be delivered face to face or remotely, in one-to-one or group settings. Where people do not respond to low intensity therapy or their problems are more complex at referral, individuals are commonly offered high intensity therapies (>=16-20 sessions), which are formulation driven one-to-one cognitive behavioural or other types of evidence-based therapies. Interventions are standardized and delivered by trained practitioners and psychotherapists following evidence-based protocols (20).

|  | **Condition** | **Psychological therapies** | **Source** |
| --- | --- | --- | --- |
| Step 2: Low-intensity interventions | Depression | Individual guided self-help based on cognitive behavioural therapy (CBT), computerised CBT, behavioural activation, structured group physical activity programme | NICE guidelines CG90, CG91, CG123 |
|  | Generalised anxiety disorder | Self-help, or guided self-help based on CBT, psycho-educational groups, computerised CBT | NICE guidelines CG113, CG123 |
|  | Panic disorder | Self-help, or guided self-help based on CBT, psycho-educational groups, computerised CBT | NICE guidelines CG31, CG123 |
|  | Obsessive-compulsive disorder | Guided self-help based on CBT | NICE guidelines CG31, CG123 |
| Step 3: High intensity interventions | Depression  For individuals with mild to moderate severity who have not responded to initial low-intensity interventions | CBT (individual or group) or interpersonal therapy (IPT)  Behavioural Activation  Couple therapy  Counselling for depression  Brief psychodynamic therapy  Note: Psychological interventions can be provided in combination with antidepressant medication | NICE guidelines: CG90, CG91, CG123 |
|  | Depression  Moderate to severe | CBT (individual) or IPT, each with medication |  |

Source: National Collaborating Centre for Mental Health. The Improving Access to Psychological Therapies Manual 2021.

Supplement B - Sensitivity analyses - Adjusted hazard ratios for cardiovascular outcomes associated with reliable recovery from depression and change in depressive symptoms scores

| Main predictor of interest  Outcome | Model 1  HR (95% CI) | p-value | Model 2  HR (95% CI) | | p-value | Model 3  HR (95% CI) | p-value |
| --- | --- | --- | --- | --- | --- | --- | --- |
|  |  |  |  | |  |  |  |
| Reliable Recovery (Yes vs No) |  |  |  | |  |  |  |
| All CV events | 0.90 (0.88; 0.91) | <.001 | 0.83 (0.81; 0.84) | | <.001 | 0.88 (0.86; 0.89) | <.001 |
| CHD | 0.84 (0.82; 0.87) | <.001 | 0.80 (0.78; 0.83) | | <.001^(a)^ | 0.88 (0.85; 0.90) | <.001^(b)^ |
| Stroke | 0.94 (0.88; 1.00) | <.001 | 0.86 (0.81; 0.92) | | <.001 | 0.91 (0.85; 0.97) | 0.009 |
| Death from any cause | 0.82 (0.80; 0.86) | <.001 | 0.74 (0.71; 0.76) | | <.001^(c)^ | 0.79 (0.76; 0.82) | <.001^(c)^ |
|  |  |  |  | |  |  |  |
| PHQ-9 Change (/6 point decrease) |  |  |  | |  |  |  |
| All CV events | 0.95 (0.94; 0.96) | <.001 | 0.94 (0.93; 0.95) | | <.001 | 0.93 (0.92; 0.94) | <.001 |
| CHD | 0.95 (0.94; 0.96) | <.001 | 0.94 (0.93; 0.96) | | <.001^(a)^ | 0.94 (0.92; 0.95) | <.001^(b)^ |
| Stroke | 0.97 (0.94; 0.99) | 0.033 | 0.95 (0.93; 0.98) | | <.001 | 0.94 (0.92; 0.98) | <.001 |
| Death from any cause | 0.90 (0.89; 0.92) | <.001 | 0.89 (0.87; 0.90) | | <.001^(c)^ | 0.89 (0.87; 0.90) | <.001^(c)^ |
|  |  |  |  | |  |  |  |
|  |  |  |  | |  |  |  |
| Model 1: main predictor of interest only  Model 2: Model 1 + demographic covariates (age, gender, ethnicity, IMD quintile)  Model 3: Model 2 + clinical covariates (baseline PHQ-9 and GAD-7 scores, psychotropic medications, long-term health condition, diabetes or hypertension at baseline, reason for ending treatment, year of appointment, number of sessions  Stratification due to non-proportional hazards for individual covariates: | | | |  |  |  |  |

1. Baseline hazard stratified by age group
2. Baseline hazard stratified by age group and PHQ category
3. Baseline hazard stratified by age group and IMD quintile

Supplement C - Sensitivity analyses - Adjusted hazard ratios for cardiovascular outcomes associated with therapy depression outcomes starting the observation period two years after end of treatment

| Study Cohort (N=510, 973)  Main predictor of interest | Model 1  HR (95% CI) | p-value | Model 2  HR (95% CI) | | p-value | Model 3  HR (95% CI) | p-value |
| --- | --- | --- | --- | --- | --- | --- | --- |
|  |  |  |  | |  |  |  |
| Reliable Improvement (Yes vs No) |  |  |  | |  |  |  |
| All CV events | 0.90 (0.89; 0.93) | <.001 | 0.87 (0.85; 0.89) | | <.001 | 0.88 (0.86; 0.90) | <.001 |
| CHD | 0.88 (0.85; 0.91) | <.001 | 0.87 (0.84; 0.90) | | <.001 | 0.89 (0.86; 0.93) | <.001 |
| Stroke | 0.89 (0.82; 0.95) | <.001 | 0.85 (0.78; 0.91) | | <.001 | 0.86 (0.79; 0.93) | <.001 |
| Death from any cause | 0.82 (0.79; 0.85) | <.001 | 0.77 (0.74; 0.80) | | <.001^(a)^ | 0.80 (0.77; 0.84) | <.001^(a)^ |
|  |  |  |  | |  |  |  |
| Reliable Recovery (Yes vs No) |  |  |  | |  |  |  |
| All CV events | 0.90 (0.88; 0.92) | <.001 | 0.84 (0.82; 0.86) | | <.001 | 0.88 (0.86; 0.90) | <.001 |
| CHD | 0.86 (0.83; 0.89) | <.001 | 0.83 (0.80; 0.85) | | <.001 | 0.89 (0.86; 0.93) | <.001 |
| Stroke | 0.92 (0.85; 0.99) | 0.034 | 0.84 (0.78; 0.91) | | <.001 | 0.90 (0.83; 0.98) | 0.012 |
| Death from any cause | 0.84 (0.80; 0.88) | <.001 | 0.75 (0.72; 0.79) | | <.001^(a)^ | 0.80 (0.77; 0.85) | <.001^(a)^ |
|  |  |  |  | |  |  |  |
| PHQ-9 Change (/6 point decrease) |  |  |  | |  |  |  |
| All CV events | 0.96 (0.95; 0.97) | <.001 | 0.94 (0.93; 0.95) | | <.001 | 0.93 (0.92; 0.94) | <.001 |
| CHD | 0.95 (0.94; 0.97) | <.001 | 0.95 (0.93; 0.96) | | <.001 | 0.94 (0.93; 0.96) | <.001 |
| Stroke | 0.97 (0.94; 1.00) | 0.081 | 0.96 (0.92; 0.99) | | 0.009 | 0.95 (0.92; 0.99) | 0.006 |
| Death from any cause | 0.91 (0.89; 0.92) | <.001 | 0.89 (0.87; 0.91) | | <.001^(a)^ | 0.89 (0.87; 0.91) | <.001^(a)^ |
|  |  |  |  | |  |  |  |
|  |  |  |  | |  |  |  |
| Model 1: main predictor of interest only  Model 2: Model 1 + demographic covariates (age, gender, ethnicity, IMD quintile)  Model 3: Model 2 + clinical covariates (baseline PHQ-9 and GAD-7 scores, psychotropic medications, long-term health condition, diabetes or hypertension at baseline, reason for ending treatment, year of appointment, number of sessions  Stratification due to non-proportional hazards for individual covariates: | | | |  |  |  |  |

1. Baseline hazard stratified by age group and IMD quintile

Supplement D - Sensitivity analyses - Adjusted hazard ratios for cardiovascular outcomes associated with therapy depression outcomes, measuring depression symptoms improvement at the last course of IAPT treatment

| Study Cohort (N=617, 510)  Main predictor of interest | Model 1  HR (95% CI) | p-value | Model 2  HR (95% CI) | p-value | Model 3  HR (95% CI) | p-value |  |
| --- | --- | --- | --- | --- | --- | --- | --- |
|  |  |  |  |  |  |  |  |
| Reliable Improvement (Yes vs No) |  |  |  |  |  |  |  |
| All CV events | 0.89 (0.87; 0.91) | <.001 | 0.85 (0.84; 0.87) | <.001 | 0.87 (0.85; 0.89) | <.001 |  |
| CHD | 0.86 (0.84; 0.89) | <.001 | 0.85 (0.83; 0.87) | <.001 | 0.88 (0.85; 0.91) | <.001^(b)^ |  |
| Stroke | 0.88 (0.82; 0.93) | <.001 | 0.85 (0.78; 0.89) | <.001 | 0.85 (0.79; 0.92) | <.001 |  |
| Death from any cause | 0.80 (0.78; 0.83) | <.001 | 0.76 (0.74; 0.79) | <.001^(a)^ | 0.80 (0.77; 0.83) | <.001^(a)^ |  |
|  |  |  |  |  |  |  |  |
| Reliable Recovery (Yes vs No) |  |  |  |  |  |  |  |
| All CV events | 0.88 (0.87; 0.90) | <.001 | 0.82 (0.80; 0.84) | <.001 | 0.87 (0.85; 0.89) | <.001 |  |
| CHD | 0.84 (0.81; 0.86) | <.001 | 0.80 (0.77; 0.83) | <.001 | 0.88 (0.85; 0.91) | <.001 |  |
| Stroke | 0.91 (0.87; 0.97) | <.001 | 0.83 (0.78; 0.89) | <.001 | 0.88 (0.82; 0.91) | <.001 |  |
| Death from any cause | 0.80 (0.77; 0.83) | <.001 | 0.72 (0.69; 0.74) | <.001^(a)^ | 0.77 (0.75; 0.81) | <.001^(a)^ |  |
|  |  |  |  |  |  |  |  |
| PHQ-9 Change (/6 point decrease) |  |  |  |  |  |  |  |
| All CV events | 0.95 (0.94; 0.96) | <.001 | 0.94 (0.93; 0.95) | <.001 | 0.93 (0.92; 0.94) | <.001 |  |
| CHD | 0.84 (0.93; 0.95) | <.001 | 0.94 (0.93; 0.95) | <.001^(b)^ | 0.93 (0.92; 0.95) | <.001^(b)^ |  |
| Stroke | 0.95 (0.93; 0.98) | <.001 | 0.94 (0.91; 0.97) | <.001 | 0.93 (0.90; 0.96) | <.001 |  |
| Death from any cause | 0.90 (0.88; 0.91) | <.001 | 0.88 (0.87; 0.89) | <.001^(a)^ | 0.88 (0.86; 0.89) | <.001^(a)^ |  |
|  |  |  |  |  |  |  |  |
|  |  |  |  |  |  |  |  |
| Model 1: main predictor of interest only  Model 2: Model 1 + demographic covariates (age, gender, ethnicity, IMD quintile)  Model 3: Model 2 + clinical covariates (baseline PHQ-9 and GAD-7 scores, psychotropic medications, long-term health condition, diabetes or hypertension at baseline, reason for ending treatment, year of appointment, number of sessions and an indicator for having previously attended IAPT  Stratification due to non-proportional hazards for individual covariates: | | | | | | | |

1. Baseline hazard stratified by age group and IMD quintile
2. Baseline hazard stratified by age group

Supplement E - Interaction tests and adjusted Hazard Ratios by age stratum in All CV events fully adjusted model

| **Interaction test**  **Sub-group** | **Adjusted HR for each subgroup (95% CI), outcome = all CV**  **Reliable improvement vs No reliable improvement** | | | |
| --- | --- | --- | --- | --- |
| *Age category * Reliable Improvement indicator interaction, global test Wald χ2 (8, N = 636, 955) =36.7, p < .001* | | | | |
|  |  |  |  | |
|  |  |  | **Further Wald tests for linear combinations of regression parameters** | |
|  |  |  | Simple test vs ref. category  *χ2 (1, N = 636, 955)* | Composite Test |
| 45-49 | 0.84 (0.81; 0.88) | <.001 | Ref | **Test that HR in age category 45-49 = 50-54 = 55-60** |
| 50-54 | 0.83 (0.80; 0.86) | <.001 | p=0 .640 | *χ2 (2, N = 636, 955)=1.25*  *p=0.5346* |
| 55-60 | 0.86 (0.82; 0.89) | <.001 | p=0.555 |  |
| 60-64 | 0.94 (0.90; 0.99) | 0.014 | p <.001 | **Test that HR in age category 60-64 = 65-69 = 70-74 = 75-79 = 80-84 = 85+**  *χ2 (5, N = 636, 955)=5.00*  *p=0.4159* |
| 65-69 | 0.90 (0.86; 0.95) | <.001 | p <0.068 |  |
| 70-74 | 0.91 (0.86; 0.98) | 0.007 | p=0.041 |  |
| 75-79 | 0.99 (0.92, 1.07) | 0.793 | p=<.001 |  |
| 80-84 | 0.93 (0.84; 1.04) | 0.199 | p=0.090 |  |
| 85+ | 0.98 (0.83; 1.15) | 0.804 | p=0.081 |  |
|  |  |  |  |  |
| *Gender * Reliable Improvement indicator interaction, global test Wald χ2 (2, N = 636, 955) =0.28 p=0.5951* | | | | |
| Female | 0.88 (0.86; 0.90) | <.001 |  |  |
| Male | 0.89 (0.86; 0.91) | <.001 |  |  |
| *Ethnicity * Reliable Improvement indicator interaction, global test χ2 (6, N = 636, 955) =6.13 p=0.4088* | | | | |
| White | 0.88 (0.86; 0.90) | <.001 |  |  |
| Mixed | 0.88 (0.73; 1.07) | 0.207 |  |  |
| Asian | 0.94 (0.84; 1.05) | 0.252 |  |  |
| Black | 0.84 (0.74; 0.95) | 0.008 |  |  |
| Chinese | 0.60 (0.31; 1.20) | 0.153 |  |  |
| Other | 1.05 (0.85; 1.28) | 0.668 |  |  |
| Missing | 0.89 (0.85; 0.94) | <.001 |  |  |
|  |  |  |  |  |
| PHQ category* Improvement indicator interaction – global test χ^2^(2*, N = 636, 955*) =1.97, p=0.3729 | | | | |
| Mild (< 10) | 0.90 (0.87; 0.93) | <.001 |  |  |
| Moderate | 0.87 (0.84; 0.90) | <.001 |  |  |
| Severe | 0.88 (0.85; 0.90) | <.001 |  |  |
|  |  |  |  |  |
| Psychotropic medications before treatment* Improvement indicator interaction – global test χ^2^(2*, N = 636, 955*) =2.73, p=0.0982 | | | | |
| Yes | 0.90 (0.87; 0.93) | <.001 |  |  |
| No | 0.87 (0.85; 0.90) | <.001 |  |  |
| Missing | 0.85 (0.81; 0.90) | <.001 |  |  |
|  |  |  |  |  |
| Long-term Health Condition before treatment* Improvement indicator interaction – global test χ^2^(2*, N = 636, 955*) =0.16, p=0.9240 | | | | |
| Yes | 0.88 (0.86; 0.91) | <.001 |  |  |
| No | 0.88 (0.85; 0.91) | <.001 |  |  |
| Missing | 0.88 (0.85; 0.91) | <.001 |  |  |
|  |  |  |  |  |
| Hypertension before treatment*Improvement indicator interaction – – global test χ^2^(1*, N = 636, 955*) =0.12, p=0.7241 | | | | |
| Yes | 0.88 (0.84; 0.91) | <.001 |  |  |
| No | 0.88 (0.86; 0.89) | <.001 |  |  |
|  |  |  |  |  |
| Diabetes before treatment*Improvement indicator interaction – – global test χ^2^(1*, N = 636, 955*) =0.12, p=0.7241 | | | | |
| Yes | 0.89 (0.83; 0.95) | <.001 |  |  |
| No | 0.88 (0.86; 0.89) | <.001 |  |  |
|  |  |  |  |  |
| Reason for ending treatment* Improvement indicator interaction global test χ^2^(5*, N = 636, 955*) =2.01, p=0.85 | | | | |
| Completed | 0.88 (0.85, 0.91) | <.001 |  |  |
| Drop-out | 0.87 (0.83; 0.92) | <.001 |  |  |
| Not suitable | 0.96 (0.77; 1.20) | 0.745 |  |  |
| Declined | 0.90 (0.79; 1.03) | 0.141 |  |  |
| Referred on | 0.93 (0.83; 1.05) | 0.243 |  |  |
| Missing | 0.88 (0.86; 0.91) | <.001 |  |  |
| \| Model 3: Time to all CV events = reliable improvement + demographic covariates (age category, gender, ethnicity, IMD quintile) + clinical covariates (baseline PHQ-9 and GAD-7 scores, psychotropic medications, long-term health condition, diabetes or hypertension at baseline, reason for ending treatment, year of appointment, number of sessions + interaction between sub-group and reliable improvement indicator \| \| --- \| | | | | |

Supplement F - Demographic and baseline characteristics by age stratum

| Baseline characteristic |  | | Age <60 | |  | | Age>=60 |  |
| --- | --- | --- | --- | --- | --- | --- | --- | --- |
|  | Overall  (N=478, 864) | With Reliable Improvement  (N=273, 876) | | Without reliable Improvement  (N=204, 988) | | Overall  (N=158 091) | With Reliable Improvement  (N=99, 747) | Without Reliable Improvement  (N=58, 334) |
|  |  |  | |  | |  |  |  |
| Age, years |  |  | |  | |  |  |  |
| Mean (SD) | 51.2 (4.2) | 51.3 (4.2) | | 51.2 (4.2) | | 66.5 (5.9) | 66.6 (5.9) | 66.3 (6.0) |
| Range | 45-59 | 45-59 | | 45-59 | | 60-101 | 60-101 | 60-101 |
|  |  |  | |  | |  |  |  |
| Gender (%) |  |  | |  | |  |  |  |
| Male | 35.5 | 35.1 | | 35.1 | | 30.1 | 30.6 | 31.6 |
| Female | 64.5 | 64.9 | | 64.9 | | 69.0 | 69.4 | 63.9 |
|  |  |  | |  | |  |  |  |
| Ethnicity (%) |  |  | |  | |  |  |  |
| White | 82.5 | 84.1 | | 80.2 | | 86.3 | 87.4 | 84.4 |
| Mixed | 1.3 | 1.2 | | 1.5 | | 0.6 | 0.5 | 0.7 |
| Asian | 3.3 | 2.9 | | 3.9 | | 2.3 | 2.0 | 2.7 |
| Black | 2.9 | 2.7 | | 3.1 | | 1.2 | 1.1 | 1.3 |
| Chinese | 0.1 | 0.1 | | 0.2 | | 0.1 | 0.1 | 0.1 |
| Other | 1.0 | 0.8 | | 1.3 | | 0.7 | 0.6 | 0.9 |
| Missing | 8.9 | 8.2 | | 9.9 | | 8.9 | 8.3 | 9.9 |
|  |  |  | |  | |  |  |  |
| IMD Quintiles (%) |  |  | |  | |  |  |  |
| 1st | 22.5 | 19.7 | | 26.3 | | 16.4 | 15.2 | 18.7 |
| 2nd | 21.3 | 20.6 | | 22.4 | | 19.3 | 18.9 | 20.0 |
| 3rd | 20.2 | 20.7 | | 19.4 | | 21.4 | 21.8 | 20.8 |
| 4th | 18.8 | 20.1 | | 17.1 | | 21.7 | 22.2 | 21.0 |
| 5th | 17.2 | 19.0 | | 14.9 | | 21.1 | 22.1 | 19.5 |
|  |  |  | |  | |  |  |  |
| PHQ-9 (Mean, SD) |  |  | |  | |  |  |  |
| Before Treatment | 17.8 (4.6) | 18.0 (4.4) | | 17.4 (4.8) | | 16.5 (4.5) | 16.9 (4.4) | 15.9 (4.6) |
| End of Treatment | 10.7 (7.2) | 6.3 (4.4) | | 16.7 (5.7) | | 8.8 (6.6) | 5.2 (4.0) | 14.9 (5.7) |
| Change (decrease) | 7.0 (6.8) | 11.8 (4.3) | | 0.7 (3.4) | | 7.7 (6.5) | 11.7 (4.2) | 1.0 (3.4) |
|  |  |  | |  | |  |  |  |
| GAD-7 (Mean,SD) |  |  | |  | |  |  |  |
| Before Treatment | 14.7 (4.5) | 14.8 (4.4) | | 14.7 (4.6) | | 13.9 (4.7) | 14.1 (4.6) | 13.7 (4.9) |
| End of Treatment | 9.1 (6.2) | 5.7 (4.3) | | 13.7 (5.2) | | 7.7 (5.8) | 5.0 (4.03) | 12.4 (5.4) |
| Change | 5.6 (6.1) | 2.3 (1.2) | | 5.7 (4.6) | | 6.2 (6.0) | 2.3 (1.2) | 0.3 (1.1) |
|  |  |  | |  | |  |  |  |
| Psychotropic medication (%) |  |  | |  | |  |  |  |
| Yes | 55.2 | 54.6 | | 56.1 | | 53.1 | 53.7 | 52.1 |
| No | 34.3 | 35.8 | | 32.3 | | 37.1 | 37.2 | 36.7 |
| Missing | 10.5 | 9.6 | | 11.6 | | 9.9 | 9.1 | 11.1 |
|  |  |  | |  | |  |  |  |
| Diabetes before treatment (%) | 3.5 | 3.2 | | 3.9 | | 6.2 | 5.9 | 6.6 |
| Hypertension before treatment (%) | 7.5 | 7.2 | | 7.9 | | 18.1 | 18.1 | 18.1 |
|  |  |  | |  | |  |  |  |
| Long-term Health Condition (%) |  |  | |  | |  |  |  |
| Yes | 28.9 | 27.1 | | 43.9 | | 36.7 | 35.9 | 38.0 |
| No | 48.0 | 51.1 | | 31.2 | | 41.0 | 42.9 | 37.8 |
| Missing | 23.2 | 21.8 | | 24.9 | | 22.3 | 21.2 | 24.2 |
|  |  |  | |  | |  |  |  |
| Reason for Treatment End (%) |  |  | |  | |  |  |  |
| Completed | 45.0 | 57.8 | | 28.0 | | 51.6 | 62.2 | 33.4 |
| Dropout | 16.7 | 11.3 | | 24.0 | | 13.3 | 9.0 | 20.1 |
| Not Suitable | 0.9 | 0.3 | | 1.7 | | 1.0 | 0.5 | 1.9 |
| Declined | 2.4 | 1.4 | | 3.6 | | 2.4 | 1.4 | 4.1 |
| Referred | 3.3 | 1.5 | | 5.6 | | 2.7 | 1.3 | 5.1 |
| Missing | 31.8 | 27.8 | | 37.1 | | 29.1 | 25.7 | 35.1 |
|  |  |  | |  | |  |  |  |
| Number of Sessions (Mean, SD) | 6.7 (4.8) | 7.5 (4.8) | | 5.7 (4.6) | | 6.4 (4.3) | 6.9 (4.2) | 5.5 (4.2) |
|  |  |  | |  | |  |  |  |
